# MicroRNA-23a-3p Inhibits Mucosal Melanoma Growth and Progression through Targeting Adenylate Cyclase 1 and Attenuating cAMP and MAPK Pathways: Erratum

**DOI:** 10.7150/thno.72945

**Published:** 2022-04-26

**Authors:** Meng Ma, Jie Dai, Huan Tang, Tianxiao Xu, Sifan Yu, Lu Si, Chuanliang Cui, Xinan Sheng, Zhihong Chi, Lili Mao, Xiaowen Wu, Lu Yang, Huan Yu, Siming Li, Bin Lian, Bixiang Tang, Xuan Wang, Xieqiao Yan, Xue Bai, Li Zhou, Yan Kong, Jun Guo

**Affiliations:** 1 Key Laboratory of Carcinogenesis and Translational Research (Ministry of Education/Beijing), Department of Renal Cancer and Melanoma, Peking University Cancer Hospital and Institute, Beijing 100142, China; 2 Department of Radiotherapy, Beijing Chest Hospital, Capital Medical University, Beijing Tuberculosis and Thoracic Tumor Research Institute, Beijing 101149, China.

In the initially published version of this article, the H&E image of Lenti-pMIR-23a group in Figure 4H was wrongly attached. We replaced the H&E picture with a correct raw data image and provide a new Figure 4 shown below. The issue described above does not affect the interpretation of the data nor conclusion of this research study. The authors apologize for any inconvenience that this error may have caused.

## Figures and Tables

**Figure 4:**
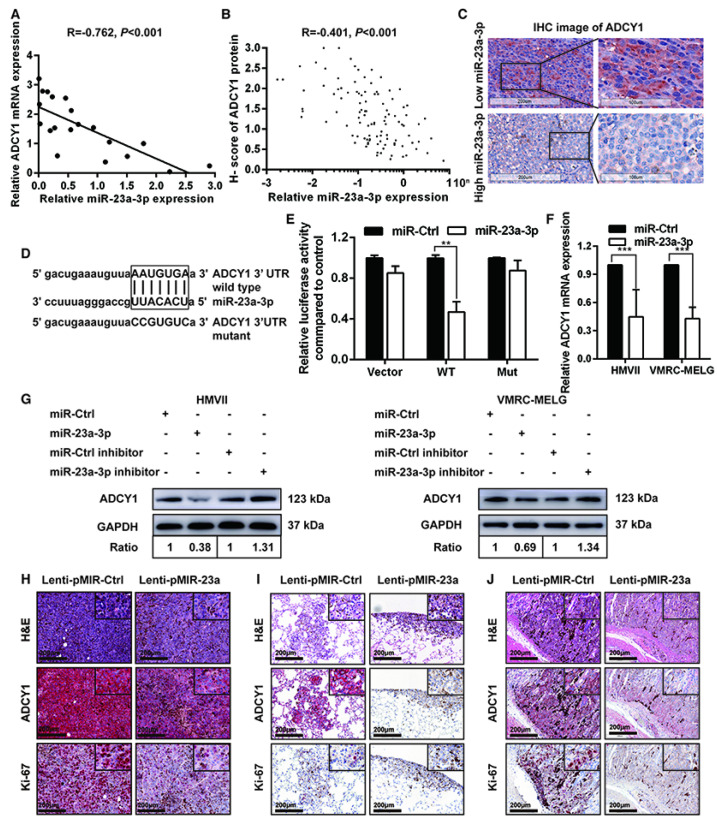
ADCY1 is a direct target of miR-23a-3p. (A) Correlation between ADCY1 mRNA and miR-23a-3p in 20 fresh-frozen MM tissues. Expression of ADCY1 mRNA and miR-23a-3p was measured by qRT-PCR and was normalized to the mean level in three normal mucosal nevi. GAPDH and U6 were used as endogenous controls, respectively. (B) Correlation between ADCY1 protein and miR-23a-3p in 117 FFPE samples. (C) Representative IHC staining images of ADCY1 in patients with low and high miR-23a-3p expression. Bar, 100 mm. (D) Putative miR-23a-3p interaction sites within the 3'-UTR of ADCY1 mRNA are indicated by boxes. Mutations were generated in the complementary site of the ADCY1 3´-UTR for the seed sequences of miR-23a-3p as shown in capital letters. (E) The full length of ADCY1 3'-UTR with putative wild-type (WT) miR-23a-3p-binding site or a binding site mutated at the 3'-UTR region (Mut) were cloned downstream of the CMV promoter in the pMIR-REPORT vector. Luciferase activity was measured after co-transfection of the reporter constructs with WT- or Mut-interacting sites of ADCY1 and miR-23a-3p mimic or miR-Ctrl in HEK293T cells. Data are presented as the mean ± SD of three independent experiments. (F) ADCY1 transcripts level at 48 h after transfection with miR-23a-3p and control as determined by qRT-PCR. GAPDH was used as an internal control. (G) HMVII and VMRC-MELG cells were transfected with miR-23a-3p mimic or inhibitor and the corresponding controls. ADCY1 protein expression after 48 h of transfection was detected by western blot analysis. Band intensities were quantified using ImageJ. (H-J) Tissue sections from subcutaneous xenografts (H), lung metastasis (I), and intra-abdominal metastasis (J) were stained with H&E, ADCY1 and Ki-67. Bar, 50 μm. *P < 0.05, **P < 0.01, ***P < 0.001.
